# Biomarkers of HIV Susceptibility and Disease Progression^☆^

**DOI:** 10.1016/j.ebiom.2014.11.003

**Published:** 2014-11-07

**Authors:** Vanessa Pirrone, Joshua Mell, Benjamin Janto, Brian Wigdahl

**Affiliations:** a Department of Microbiology and Immunology, Institute for Molecular Medicine and Infectious Disease, Drexel University College of Medicine, Philadelphia, PA 19102, United States; b Center for Molecular Virology and Translational Neuroscience, Institute for Molecular Medicine and Infectious Disease, Drexel University College of Medicine, Philadelphia, PA 19102, United States; c Center for Genomic Sciences, Institute for Molecular Medicine and Infectious Disease, Drexel University College of Medicine, Philadelphia, PA 19102, United States

Both host and viral factors are associated with HIV-1 susceptibility and disease progression. Host factors can typically be divided into three categories (Chatterjee, 2010): 1) genes encoding cell-surface receptors or ligands for these proteins, including the classic CCR5 32-bp deletion; 2) genes within the human leukocyte antigens (HLA) that regulate host responses to infection; and 3) other cytokine and immune response genes, including the homozygosity at position − 308 in the TNF-alpha promoter. More recently genome-wide association studies (GWAS) have detected new genetic factors associated with HIV-1 infection (van Manen et al., 2012). In addition, an extensive array of host physiological responses have been assessed, including MCP-1 (CCL2) (Price et al., 2007).

In relation to HIV-1 susceptibility, the co-receptors CXCR4 and CCR5 are classically linked to infection of T cells and cells of the monocyte–macrophage lineage (MML), respectively. CCR5-utilizing viruses are the major type of virus transmitted therefore MML cells have been thought to contribute to early disease progression. More recently it has been demonstrated that T lymphocytes may also be a major vehicle for HIV-1 transmission. Transmitted viruses preferentially use CCR5 as a co-receptor, require high levels of CD4, and are able to replicate efficiently in primary CD4 + T-cells, suggesting a CCR5-utilizing T-tropic viral phenotype (Ochsenbauer et al., 2012). Subsequently, these founder viruses can infect MML cells, resulting in the development of a wider target cell population that expresses higher levels of CCR5 than T cells (Ochsenbauer et al., 2012). Although in the classical sense, co-receptor utilization remains the most important factor in HIV-1 susceptibility, a number of other host factors are currently under investigation that also appear to play a role in cellular susceptibility. Often these host factors interact with viral proteins thereby affecting the efficiency of viral infection.

HIV-1 viral protein R (Vpr) is a multifunctional virion-associated accessory protein (Cohen et al., 1990). Vpr functions both early after entry into cells and also following integration of the proviral genome (Hrimech et al., 1999). Vpr is a component of the preintegration complex, and following nuclear import Vpr likely plays a major role in regulating the expression of immediate-early HIV-1 genes from the now integrated proviral genome. This mechanism is utilized prior to the transition to Tat-driven gene expression. Vpr is critical in the viral replication cycle in non-dividing cells, such as monocytes (Connor et al., 1995), whereas it has been shown to be dispensable in T-lymphocytes (Eckstein et al., 2001).

In the manuscript by Chiang et al. within this issue of *EBioMedicine*, a new host factor is described as playing a role in HIV-1 susceptibility (Chiang et al., 2014). This host factor, MRJ-L, is the large splice isoform of DNAJB6 (a homolog of heat shock protein 40) and the authors state that it has been shown to interact with Vpr, however this data, to date has not been published and therefore this point is in need of further detailed exploration. The authors, studying cohorts of “men who had sex with men” (MSM), found that macrophage expression of MRJ-L varied among healthy subjects but was significantly and substantially higher in HIV-1-infected subjects. The macrophage MRJ-L expression level predicted the risk of HIV-1 infection with similar power as whether or not subjects engaged in unprotected sexual contact. To demonstrate a cause-and-effect relationship between MRJ-L expression and HIV-1 susceptibility, monocytes from healthy subjects were isolated and then differentiated and infected ex vivo. Interestingly, this experiment revealed that macrophages with high MRJ-L expression were more susceptible to HIV-1 infection than those with low MRJ-L expression. Furthermore, RNAi knockdown of MRJ-L in high-expressing cells decreased susceptibility to infection, while lentiviral over-expression of MRJ-L in low-expressing cells increased their susceptibility to infection. These results clearly implicate high expression of MRJ-L with macrophage susceptibility to HIV infection. The authors were able to identify a plausible mechanism, showing that MRJ-L colocalizes with Vpr and is required for localizing Vpr to the nucleus. Although the localization experiments were performed in HeLa cells, because of the importance of Vpr in macrophages, the extension can be made that MRJ-L plays a major role in the nuclear transport of this important viral protein (and possibly the whole pre-integration complex) in other cell types. The stochastic expression of MRJ-L in uninfected subjects raises the possibility that genetic factors controlling this variability are also responsible for variability in susceptibility to infection.

Numerous studies have demonstrated the role of host proteins in HIV-1 susceptibility, and the strong evidence provided by Chiang et al. that MRJ-L levels impact susceptibility to infection makes it an attractive therapeutic target. However, caution needs to be taken with regard to developing a therapy to reduce MRJ-L expression. Studies have shown a link between decreased levels of MRJ-L and malignancies such as breast cancer, with MRJ-L being shown to regulate several key players in tumor formation and metastasis, and demonstrating its ability to functionally retard tumor growth (Mitra et al., 2008). More complete studies are needed to determine the role of MRJ-L in macrophages, as well as more directed therapeutic targeting to specific cells to avoid potentially devastating off-target consequences.

## Conflicts of Interest

The authors declared no conflicts of interest.
